# Acknowledgment of *Ad Hoc* Reviewers

**DOI:** 10.1128/mSphere.00543-17

**Published:** 2017-12-27

**Authors:** Michael J. Imperiale

**Affiliations:** University of Michigan Medical School, Ann Arbor, Michigan, USA

## EDITORIAL

As we reach the end of 2017, our second full year of publishing cutting-edge research in the microbial sciences, the *mSphere* Senior Editors and Editors would like to offer our sincere thanks to the many individuals who have acted as referees of the manuscripts that we have handled this year. They have helped us continue to achieve our goal of delivering critical, rapid, and fair decisions to our authors.

Kjersti Aagaard
Robert B. Abramovitch
Christina Jane Adler
Hector C. Aguilar
Sebastian Aguirre
Tero Ahola
Jose A. Ainsa
Klaus Aktories
Alexandre Alanio
César G. Albariño
Randy A. Albrecht
Gladys Alexandre
Irving Coy Allen
Igor C. Almeida
Francis Alonzo
Ryan Altman
Linda A. Amaral-Zettler
Francisco Amaro
Katherine R. Amato
David R. Andes
Haike Antelmann
Michelle M. Arnold
Koichiro Awai
Nuno F. Azevedo
Taeok Bae
Justin Bahl
Connor Gavin George Bamford
Jeffrey A. Banas
Horia Leonard Banciu
Claudio Bandi
Anirban Banerjee
Bruce W. Banfield
James D. Bangs
Rodolphe Barrangou
Carlos Alberto Barrera
Christopher F. Basler
Alison Baylay
Bernard W. Beall
Anne Beauvais
Simon Beddows
Terrence Bell
Vivian Bellofatto
Stefan Bereswill
Teresa M. Bergholz
Kirk Bergstrom
Kristen A. Bernard
Sonja M. Best
Roby P. Bhattacharyya
Sanjib Bhattacharyya
Kyle Bibby
Richard J. Birtles
Carol D. Blair
Jaime E. Blair
Martin Blume
Subbarao Bondada
Cyril Bontemps
Irene Bosch
Jeffrey Bose
Yan Boucher
Michèle Bouloy
David Alan Boyd
Peter J. Bradley
Aaron Brault
Erhard Bremer
Michael J. Brennan
Amanda Brown
Linda Brunotte
Kendall Bryant
Michelle Monique Conni Buckner
Gertrude Case Buehring
Silvia Bulgheresi
Catharine Christine Bulik
Pete Bull
Barbara Burleigh
Alejandro Buschiazzo
Karen Bush
Sarah N. Buss
Simone Mario Caccio
Alexandra Campbell
Laura Campisi
J. Gregory Caporaso
Paul Carini
Heather Carleton
Arturo Casadevall
Fabricio Dario Cassán
Maria Belen Cassera
Byron Caughey
Rodrigo Cayo
Gary C. Chan
Jyotsna Chandra
Steve J. Charette
Remi N. Charrel
Dipankar Chatterji
Michael S. Chaussee
Tianmu Chen
Gong Cheng
Charles Y. Chiu
Ted G. Clark
Eric S. Cole
Laurie E. Comstock
John H. Connor
Cynthia Nau Cornelissen
Muriel Cornet
Jeroen Corver
Donald L. Court
Melanie T. Cushion
Christiane Dahl
Bertrand Daignan-Fornier
Steven L. Daniel
Sujoy K. Das Gupta
Kevin M. D’Auria
Philip Davidson
Dana A. Davis
Paul H. Davis
Denise Dearing
Miranda De Graaf
Boudewijn L. de Jonge
Rosa M. del Angel
Juan Carlos de la Torre
Frank R. DeLeo
Eric L. Delwart
Paul DeMott
Vincent J. Denef
Jigar V. Desai
Suzanne Devkota
Dubraska Vanessa Diaz Campos
Jennifer Dien Bard
Monika Dolejska
Rodney M. Donlan
Patricia C. Dos Santos
Adam Driks
Felix S. Dube
Jaquelin P. Dudley
Leonard R. Duncan
Anne K. Dunn
Jeffrey D. Dvorin
Paul E. Wischmeyer
Kathryn M. Edwards
Shigeki Ehira
Jeremy R. Ellermeier
Shiro Endo
Antonio Faciola
Daniel Falush
Seamus Fanning
Rhys A. Farrer
Michael J. Federle
Jamie Findlow
Steven E. Finkel
Nicole Fischer
Mark A. Fisher
Daniel P. Flaherty
Enrique Flores
Jason Folster
Larry J. Forney
Genevieve Giny Fouda
Betsy Foxman
David N. Fredricks
Nancy E. Freitag
Jean Marie A. Frere
Benoît Gamain
Celia Garcia
Erin Garcia
Shaan L. Gellatly
Caroline Attardo Genco
Maria Laura Gennaro
Joan A. Geoghegan
Benjamin Gewurz
Rohit Ghai
Tim Wolf Gilberger
Janet R. Gilsdorf
Henry P. Godfrey
Elena A. Govorkova
Anthony Griffiths
Nigel Grimsley
Hans-Peter F. Grossart
Trudy H. Grossman
Bradley Groveman
Maria-Eugenia Guazzaroni
Patricia Guerry
Benjamin G. Hale
Lauren Hale
Scott B. Halstead
Michael R. Hamblin
Lynn E. Hancock
Philip C. Hanna
Omar Harb
Hermie J. M. Harmsen
Shimon Harrus
Roland Hatzenpichler
Joshua Hayes
Qingfang He
Shaomei He
Christine Heilmann
Joseph Heitman
Christiane Herden
Matthias Hess
David S. Hibbett
Thomas Hoenen
Deborah A. Hogan
Niels Høiby
Michael Holbrook
Christopher Keith Hope
Stacy M. Horner
Alexander R. Horswill
Daniel K. Howe
Ke Hu
Jenni Hultman
Romney M. Humphries
Edward Hutchinson
Kevin Hybiske
Alexander Idnurm
Zehra Esra Ilhan
Wataru Iwasaki
Anna C. Jacobs
Steven Jacobson
Mohamed A. Jamal
Elie Jami
Armando Jardim
Ian B. Jeffery
Seok Hoon Jeong
Aaron Richard Jex
Diego Javier Jiménez
Jörgen Johansson
Jeremiah G. Johnson
David Kadosh
Bjorn Kafsack
Barbara C. Kahl
Lindsay Kalan
Joseph Kanabrocki
Dae Wook Kang
Hyun Ah Kang
Erik A. Karlsson
Sophia Kathariou
Andrew L. Kau
Saeed Khan
Todd Kitten
Bruce S. Klein
Sabine Kleinsteuber
Laura J. Knoll
Satoshi Koike
Abimbola O. Kolawole
James B. Konopka
Jeroen Kortekaas
Peter James Krause
Matthias Kretschmer
Christopher J. Kristich
Beate Mareike Kümmerer
Hiroyuki Kusada
Kyung J. Kwon-Chung
Borden Lacy
Sushmita D. Lahiri
Seema Lakdawala
Tommy Tsan-Yuk Lam
Gordon Langsley
Patrick Lee
Samuel A. Lee
Fabian Hubertus Leendertz
Megan Denise Lenardon
Fernanda Lessa
Russell E. Lewis
Xian-Zhi Li
Xiaorong Lin
Michail S. Lionakis
Olga Lomovskaya
Jose L. Lopez-Ribot
Stephen Lory
Stilianos Louca
Andrew Lee Lovering
Juan E. Ludert
Hugo D. Lujan
Hinh Ly
Jiyan Ma
Michael J. Mahan
Andrea Maisner
Teruya Maki
Rex R. Malmstrom
Evelyne Mann
Kevin Maringer
Linsey Marr
Adam Martiny
Kevin M. Mason
Wendy Maury
Larry S. McDaniel
Bradford S. McGwire
Sarah M. McLeod
Joel McManus
David G. Meckes
Edward Mee
D. Scott Merrell
Justin Merritt
Dennis W. Metzger
David K. Meyerholz
Matthew Mikoleit
Alita A. Miller
Kathryn Milligan-Myhre
Christine Miossec
Pedro Miramón
David R. Mitchell
Tanya Miura
Paul T. Monis
Eduard Monso
Martin L. Moore
Lisa A. Morici
Yuki Morono
W. Scott Moye-Rowley
Ann-Kristin Mueller
Karl Munger
Alvaro Munoz
Vasant Muralidharan
Caitlin N. Murphy
Ephantus J. Muturi
Joe S. Mymryk
Vinay Kumar Nandicoori
Carl F. Nathan
Lama Nazzal
Esther Ndungo
David E. Nelson
Jeniel E. Nett
Minh Hong Nguyen
Connie Nichols
Anthony V. Nicola
Amelia Nieto
Masaru Konishi Nobu
Eva Nohynkova
Christopher T. Nomura
Michael James Noto
Austin Nuxoll
George-John E. Nychas
James P. O’Gara
Julia Oh
Anil Ojha
Antonio Oliver
Michael Olson
Klaus Osterrieder
Marc Ouellette
Leah R. Padgett
Gustavo Palacios
Juan Carlos Palomino
Sünje Johanna Pamp
Krisztina M. Papp-Wallace
Daniel Paredes-Sabja
Alexander R. Paredez
John S. Parker
Colin R. Parrish
Marilyn Parsons
Sally R. Partridge
Jordan Peccia
Victoria Pederick
William Penwell
Rushika Perera
Daniel Perez
Michael A. Pfaller
Hang T. T. Phan
Bruno Pichon
Johann Pitout
Paul J. Planet
Michael Poulsen
Sean T. Prigge
Alexander J. Probst
Ruben Props
Michael J. Pucci
Alexandra E. Purdy
Udi Qimron
Hongmin Quin
Ralf Rabus
Fiona Radcliff
Bethany A. Rader
Srinivasan Ramakrishnan
Kumaran S. Ramamurthi
Adam J. Ratner
Timothy D. Read
Michael Reese
Matthew Reeves
Michelle Lynne Reniere
Federico Rey
Judith C. Rhodes
Kelly C. Rice
Bert K. Rima
Amariliz Rivera
Marjorie Robert-Guroff
Sally Roberts
D. Ashley Robinson
Alejandra Rodríguez-Verdugo
Antonis Rokas
Angela Römer-Oberdörfer
Adriana E. Rosato
Jason W. Rosch
David Rosen
Dennis Rubbenstroth
Larisa Rudenko
Charles J. Russell
Daniel F. Sahm
Nina R. Salama
Raquel Sa-Leao
Stephen Salipante
Doblin Sandai
Carlos A. Sariol
Pauline Deirdre Scanlan
Carolyn R. Schaeffer
Bernhard H. Schink
Hildgund Schrempf
Raymond Schuch
Stacey Schultz-Cherry
Margaret Scull
Ingrid L. Scully
David Scurr
Anna Maria Seekatz
Mohammad R. Seyedsayamdost
Kathy Ho Yen Shair
Michael Shapira
Lindsey N. Shaw
Timothy P. Sheahan
Aimee Shen
Paul Sheridan
Alaullah Shiekh
Patricia J. Simner
Steven M. Singer
Emma Skoog
Richard Slemons
Robert Smith
Magdalene Y. So
Joseph A. Sorg
Jessica R. Spengler
Katherine R. Spindler
Jason E. Stajich
Christopher Staley
David Stallknecht
Judith Steenbergen
Peter D. Steinberg
Roger Stephan
Philip Stewart
Scott Stibitz
Charles W. Stratton
Daniel Streblow
Christopher Sullivan
Yvonne Sun
Joyce A. Sutcliffe
Troy Sutton
Staffan G. Svärd
Aishwarya Swaminathan
W. Edward Swords
Christine M. Szymanski
Clifford C. Taggart
Shumin Tan
Michael E. Taveirne
Benjamin R. tenOever
Saravanan Thangamani
Alex G. Therien
Casey Theriot
Justin Adam Thornton
Hua Tian
Anna D. Tischler
Erb Tobias
Andrew Tomaras
Alfredo G. Torres
Javier Torres
Maria Rosaria Torrisi
Jonathan S. Towner
Lindsay Traeger
Wen-Yang Tsai
Masataka Tsuda
Paul M. Tulkens
Ebenezer Tumban
Paul Turner
Anne-Catrin Uhlemann
Bruce A. Vallance
Hilde M. van der Schaar
Michael L. Vasil
Emily Vogtmann
Julia A. Vorholt
Valmik Vyas
Maggie Wagner
Irene Wagner-Doebler
Kevin John Waldron
Edward Walker
David Walsh
Xiu-Feng Wan
Guangshun Wang
Matthew J. Wargo
Digby F. Warner
David W. Warnock
Jim Wassil
Keith E. Weaver
Richard J. Webby
David S. Weiss
Louis M. Weiss
Thea Whitman
Nathan P. Wiederhold
Angela Wilks
Rob J. L. Willems
Candace L. Williams
Duncan Wilson
Sharon Witonsky
Kenneth W. Witwer
Thomas K. Wood
Floyd L. Wormley, Jr.
Jianping Xu
Yoshiharu Yamaichi
Pamela Yeh
Jeremy A. Yethon
Masafumi Yohda
Elton T. Young
Fidel Zavala
Weiping Zhang
Guan Zhu
Adam Zlotnick

